# Supplementary material for: Rapid turnover of life-cycle-related genes in the brown algae
Source: Genome Biol. 2019 Feb 14;20:35. doi: 10.1186/s13059-019-1630-6 (PMC6374913; doi:10.1186/s13059-019-1630-6)
Supplement: Supplementary file 3 — Protein alignments, corresponding Gblocks html file and CDS sequences for the lineage conserved gametophyte-biased genes. (ZIP 2109 kb) [file 13059_2019_1630_MOESM3_ESM.zip › Con_GA_alignments/Ec-07_006620.aa_ali.fasta-gb.htm]

Ec-07\_006620.aa.fasta.trans.aa\_ali.fasta


## Gblocks 0.91b Results

Processed file: **Ec-07\_006620.aa.fasta.trans.aa\_ali.fasta**  
Number of sequences: **2**  
Alignment assumed to be: **Protein**  
New number of positions: **316** (selected positions are underlined in blue)

```
                         10        20        30        40        50        60
                 =========+=========+=========+=========+=========+=========+
Ec-07_006620     MRDICWWCCNLLPFMLLLLLPWIEGAAAADERPPPHGSSRRFHDPKATLYEVLGVSKHSS
MSTRG.18885.1    ------------------------------------------------------------
                                                                             


                         70        80        90       100       110       120
                 =========+=========+=========+=========+=========+=========+
Ec-07_006620     RKEIKRVYLGLARVMHPDKKGPFRNDEVASEANDIFVKIKEAYETLYDPILRQQYDLTGM
MSTRG.18885.1    ------------------------------------------------------------
                                                                             


                        130       140       150       160       170       180
                 =========+=========+=========+=========+=========+=========+
Ec-07_006620     KEGVNNMPGEEEPEERYQDAPFGIFIRFGGGRGGLSFRYKGHGKRRAPDLMVALPVTFEE
MSTRG.18885.1    -------------------------------------------------------LTFEE
                                                                         ####


                        190       200       210       220       230       240
                 =========+=========+=========+=========+=========+=========+
Ec-07_006620     AFEGKTANVTVARERLCRTCGGTGASEPGEMPTCNLCEGKGYAYHLFAVHESGTQSDHHS
MSTRG.18885.1    VFEGKTANVTVTRERLCRTCGGTGASEPEKMPKCNLCEGKGYAYHLFGVHES------ST
                 ####################################################        


                        250       260       270       280       290       300
                 =========+=========+=========+=========+=========+=========+
Ec-07_006620     HGDRHHHDDDASSGNPRGRCSEHLDGRRDGVSHG---GGGDGLPGYAHAVNTTCKVCGGT
MSTRG.18885.1    HGHKQDHHSHTFTGTPKGRCSAHEDGSNKNGSGGDRAGASPEVPGYAHAVNTTCKVCGGT
                 ##################################   #######################


                        310       320       330       340       350       360
                 =========+=========+=========+=========+=========+=========+
Ec-07_006620     GKVSDGGCPDCRGRKVKVETETFEVTLPAGVLPGHAVILVGKGIEHPDKLPGSVQVVAVQ
MSTRG.18885.1    GKISDGGCPDCRGKRVKVETDTFEVKMPAGVLPGHTVHFGGQGTQHPDKLPGSIRVVAAQ
                 ############################################################


                        370       380       390       400       410       420
                 =========+=========+=========+=========+=========+=========+
Ec-07_006620     NAHPRFERLGADLIYRKKISLIDALLGFERMLTLLDGTRVPIVHDQVTLSGYQQELPGLG
MSTRG.18885.1    LAHPRFERQGADLVYKKKISLLDALEGFERMLTMLDGSRVRIVHDEITLSGYRQELPNLG
                 ############################################################


                        430       440       450       460       470       480
                 =========+=========+=========+=========+=========+=========+
Ec-07_006620     FPVVG-----GDGDRGSLVIDFEVDFPSRLREAHLAALRVVLTEEEIALLEDVLKLMSTR
MSTRG.18885.1    FPVVGTDNGDGDGERGALIVDFEVDFPVELREDHLVALRAVLTEEEISVLEDVLKLMSAR
                 #####     ##################################################


                        490       500       510       520       530       540
                 =========+=========+=========+=========+=========+=========+
Ec-07_006620     KSK-ARPPMEPLEYLYTRICSSD----------------DE-DDFINNGSNPRCVI-DLV
MSTRG.18885.1    KAESWRPEMDPLEYRYTRFCSLEGHFGERTKVGGADGRSSKRSD-STRGINTRCCIPDLL
                 #    ################                                       


                        550       560       570       580
                 =========+=========+=========+=========+=
Ec-07_006620     WWARPQ---------------LDDDDDGSDYESYE---EFR
MSTRG.18885.1    WWARPWVSDFIDPDHDAENDFVDDDEGGGGANSDADYHPRL
                                       ###########
```

```
Parameters used
Minimum Number Of Sequences For A Conserved Position: 2
Minimum Number Of Sequences For A Flanking Position: 2
Maximum Number Of Contiguous Nonconserved Positions: 8
Minimum Length Of A Block: 10
Allowed Gap Positions: None
Use Similarity Matrices: Yes
```

```
Flank positions of the 6 selected block(s)
Flanks: [177  232]  [241  274]  [278  425]  [431  481]  [486  501]  [563  573]  

New number of positions in Ec-07_006620.aa.fasta.trans.aa_ali.fasta-gb:  316  (54% of the original 581 positions)
```
